# PTPN2 Deficiency Enhances Programmed T Cell Expansion and Survival Capacity of Activated T Cells

**DOI:** 10.1016/j.celrep.2020.107957

**Published:** 2020-07-28

**Authors:** Markus Flosbach, Susanne G. Oberle, Stefanie Scherer, Jana Zecha, Madlaina von Hoesslin, Florian Wiede, Vijaykumar Chennupati, Jolie G. Cullen, Markus List, Josch K. Pauling, Jan Baumbach, Bernhard Kuster, Tony Tiganis, Dietmar Zehn

**Affiliations:** 1 Division of Animal Physiology and Immunology, TUM School of Life Sciences Weihenstephan, Technical University of Munich (TUM), Freising, Germany; 2 Division of Immunology and Allergy, Department of Medicine, Lausanne University Hospital, Lausanne, Switzerland; 3 Chair of Proteomics and Bioanalytics, TUM School of Life Sciences Weihenstephan, Technical University of Munich (TUM), Freising, Germany; 4 Department of Biochemistry and Molecular Biology, Monash University, Clayton, VIC 3800, Australia; 5 Peter MacCallum Cancer Centre, Melbourne, VIC 3000, Australia; 6 Big Data in BioMedicine Group, Chair of Experimental Bioinformatics, TUM School of Life Sciences Weihenstephan, Technical University of Munich (TUM), Freising, Germany; 7 ZD.B Junior Research Group LipiTUM, Chair of Experimental Bioinformatics, TUM School of Life Sciences Weihenstephan, Technical University of Munich (TUM), Freising, Germany; 8 Chair of Experimental Bioinformatics, TUM School of Life Sciences Weihenstephan, Technical University of Munich (TUM), Freising, Germany; 9 Monash Biomedicine Discovery Institute, Monash University, Clayton, VIC 3800, Australia

**Keywords:** Protein tyrosine phosphatase non‑receptor type 2 (PTPN2), T cell memory, programmed T cell expansion, adoptive T cell transfer, immunotherapy, effector T cells, phosphoproteome, autoimmunity, GWAS, single point mutation

## Abstract

Manipulating molecules that impact T cell receptor (TCR) or cytokine signaling, such as the protein tyrosine phosphatase non-receptor type 2 (PTPN2), has significant potential for advancing T cell-based immunotherapies. Nonetheless, it remains unclear how PTPN2 impacts the activation, survival, and memory formation of T cells. We find that PTPN2 deficiency renders cells *in vivo* and *in vitro* less dependent on survival-promoting cytokines, such as interleukin (IL)-2 and IL-15. Remarkably, briefly *ex vivo*-activated PTPN2-deficient T cells accumulate in 3- to 11-fold higher numbers following transfer into unmanipulated, antigen-free mice. Moreover, the absence of PTPN2 augments the survival of short-lived effector T cells and allows them to robustly re-expand upon secondary challenge. Importantly, we find no evidence for impaired effector function or memory formation. Mechanistically, PTPN2 deficiency causes broad changes in the expression and phosphorylation of T cell expansion and survival-associated proteins. Altogether, our data underline the therapeutic potential of targeting PTPN2 in T cell-based therapies to augment the number and survival capacity of antigen-specific T cells.

## Highlights

• PTPN2 deletion increases the expansion and survival of effector T cells
• PTPN2-deficient CD8^+^ T cells expand 11-fold more efficiently upon activated T cell transfer
• PTPN2 deletion permits the re-expansion of T cells with a terminal effector phenotype

## Graphical Abstract

**Figure:**
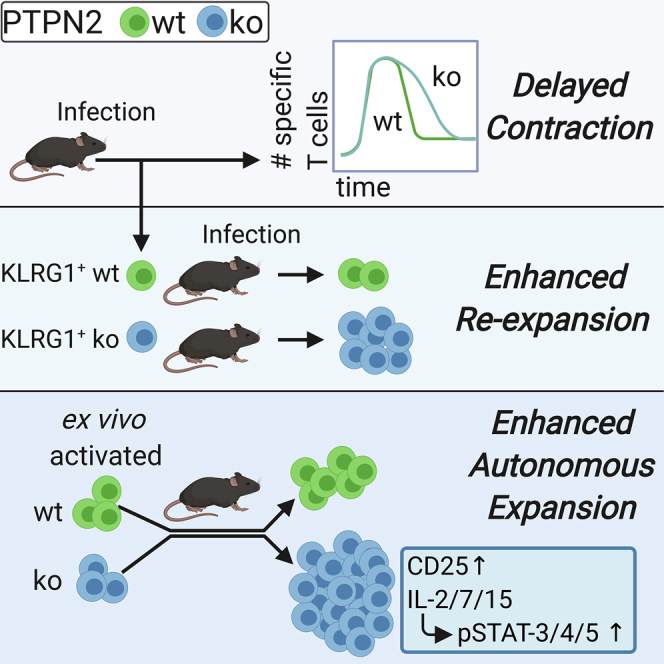

## Teaser

Manipulating PTPN2 has potential for advancing immunotherapies. However, it is unclear how PTPN2 impacts the activation, survival, and memory formation of T cells. Flosbach et al. demonstrate that PTPN2-deficient T cells are less dependent on cytokines and show increased programmed expansion, and short-lived effector T cells have enhanced survival and expansion capacity.

## Introduction

Protein tyrosine phosphatase non-receptor type 2 (PTPN2) is a ubiquitously expressed tyrosine phosphatase found at high levels in resting and activated T cells (Tiganis and Bennett, 2007; Wiede et al., 2011). Genome-wide association studies (GWASs) raised particular interest in this molecule because loss-of-function single-nucleotide polymorphisms (SNP) in PTPN2 can confer a predisposition for the development of autoimmune diseases. These include type 1 diabetes (Burton et al., 2007; Todd et al., 2007), rheumatoid arthritis, Crohn’s disease, and celiac disease (Espino-Paisan et al., 2011; Festen et al., 2011; Smyth et al., 2008).

A T cell-specific deletion of PTPN2 promotes the development of systemic inflammation and autoimmunity in otherwise non-autoimmune-prone C57BL/6 mice and accelerates the onset of type 1 diabetes in autoimmune-prone non-obese diabetic (NOD) mice (Wiede et al., 2011, 2019; Zikherman and Weiss, 2011). Mechanistically, PTPN2 has been shown to directly dephosphorylate Lck and Fyn, two major mediators of T cell receptor (TCR) signaling in CD4 and CD8 T cells (Wiede et al., 2011; Zikherman and Weiss, 2011). PTPN2 deficiency in T cells leads to increased antigen sensitivity *in vitro* and *in vivo*. This enhances the proliferative potential of T cells upon encountering antigen, as well as their capacity to undergo homeostatic proliferation, and favors the development of autoreactive T cells (Goldrath et al., 2004; Wiede et al., 2011, 2014a, 2019). Additionally, PTPN2 is known to attenuate cytokine signaling via dephosphorylation of Janus-activated kinases (JAK)-1 and -3 and signal transducers and activators of transcription (STAT)-1, -3, -5, and -6 in a cell context-dependent manner (Fukushima et al., 2010; ten Hoeve et al., 2002; Loh et al., 2011; Lu et al., 2007; Shields et al., 2013; Simoncic et al., 2002; Tiganis and Bennett, 2007; Yamamoto et al., 2002). The inflammation and spontaneous autoimmunity caused by PTPN2 deficiency in T cells can therefore be attributed to self-antigen-mediated increases in TCR sensitivity and the enhanced responses to common γ-chain cytokines (Fukushima et al., 2010; Wiede et al., 2011, 2014a; Zikherman and Weiss, 2011). Despite these documented and wide-ranging effects of PTPN2 on T cell signaling in the context of autoimmunity, it remained unknown how PTPN2 affects T cell differentiation in an acute infection in terms of clonal expansion, effector function, and the formation of autoantigen- or foreign antigen-specific memory T cells (van Lier et al., 2003; Tscharke et al., 2015).

To address this, we used a well-controlled, previously established experimental system for the T cell-specific conditional deletion of PTPN2 (Wiede et al., 2011) in combination with different infection models. We found that the complete absence of PTPN2 did not affect the principal ability to generate KLRG1^+^ effector and CD127^+^ memory T cells, but it delayed the decline of KLRG1^+^ T cells during the contraction phase and promoted their re-expansion capacity. The enhanced survival was associated with increased common γ-chain cytokine signaling and a markedly increased adoptive transfer capacity of briefly *ex vivo*-activated T cells. Altogether our data underline that eliminating PTPN2 has significant potential to enhance the efficacy of T cell-based immunotherapies.

## Results

### PTPN2 Deficiency Promotes the Long-Term Maintenance of T Cells that Lack a Typical CD127^+^ Memory Phenotype

**Figure 1:**
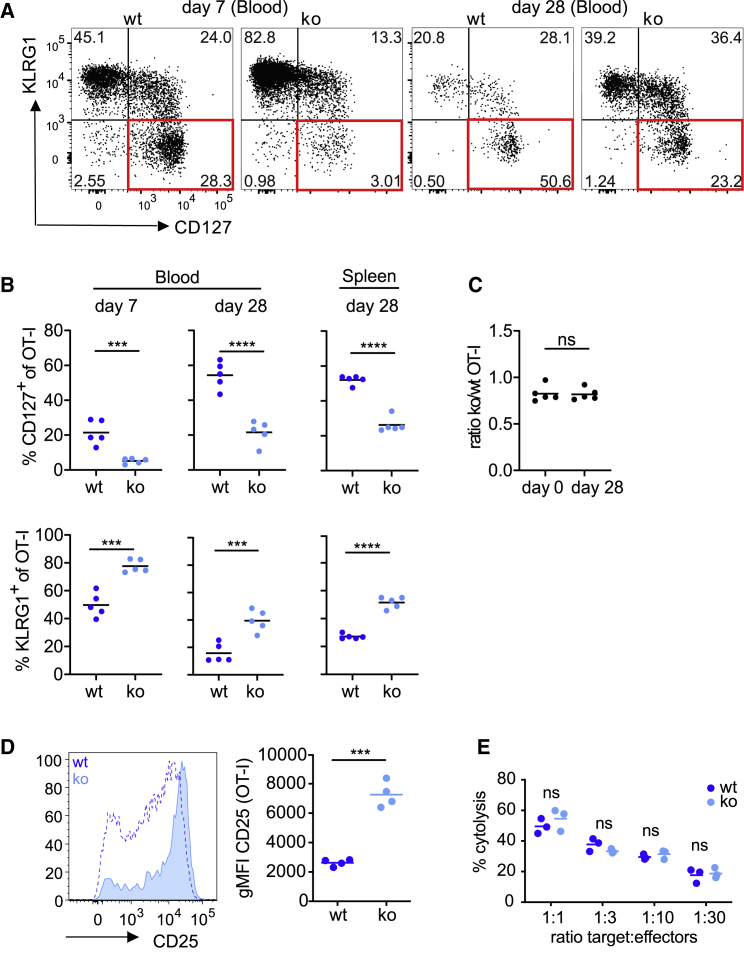
PTPN2 Alters the Ratio of Terminal Effector versus Memory Precursor T Cells CD45-congenic C57BL/6J host mice were grafted with 10^4^ WT or KO OT-I T cells and infected with 1,000 colony-forming units (CFUs) Lm-N4 24 h later. (A and B) Peripheral blood T cells were analyzed by flow cytometry at 7 and 28 days post infection (dpi) and splenic T cells at 28 days post infection. (A) The depicted flow cytometry plots are representative blood samples. (B) The dot plots show the frequencies of CD127^+^ (upper row) or KLRG1^+^ (lower row) cells within the OT-I T cell population. (C) CD45-congenic C57BL/6J host mice received 10^4^ OT-I;*Lck-*Cre;*Ptpn2*^*fl/fl*^ (KO) and OT-I;*Ptpn2*^*fl/fl*^ (WT) cells and were infected 24 h later with 1,000 CFUs Lm-N4. The dot plots show the ratio of total PTPN2-deficient versus WT T cells at the day of infection and at 28 dpi. (D) Splenic OT-I T cells were analyzed by flow cytometry for CD25 expression 4 days after infection. Representative histogram overlays of PTPN2-deficient (solid, light blue) versus WT (dotted line) OT-I T cells, and geometric mean fluorescence intensity (MFI) data for all mice are shown. (E) Splenic WT and KO OT-I T cells were isolated 7 days post infection and co-incubated with DAPI-labeled peptide-pulsed splenocytes at titrated doses for 18 h. Shown is the fraction of target cells that were lysed by WT or PTPN2-deficient OT-I T cells. The data are representative of at least two independent experiments with three to five mice in each group, and the horizontal line represents the mean. Statistical analysis: unpaired t test, ^∗∗∗∗^p ≤ 0.00001, ^∗∗∗^p ≤ 0.0001, ^ns^p ≥ 0.05. ns, not significant.

To explore how PTPN2 impacts T cell differentiation, we took advantage of TCR-transgenic mice that develop MHC class I-restricted and ovalbumin-specific CD8^+^ T cells (OT-I T cells), in which the fifth exon of PTPN2 is flanked by LoxP sites (Loh et al., 2011). T cell-specific recombination and PTPN2 inactivation were achieved through lymphocyte-specific protein tyrosine kinase (Lck)-driven expression of Cre recombinase (Wiede et al., 2011). Because this leads to the deletion of PTPN2 in the thymus at double-negative stage 3/4 (Wiede et al., 2017), we first measured whether thymic development of OT-I T cells is impacted in the absence of PTPN2. Neither the cellularity of CD4^+^CD8^+^ double-positive and CD8 single-positive T cells, nor the frequency of mature CD24^−^ cells among CD8 single-positive OT-I T cells or the CD69 expression status of double-positive OT-I T cells had changed without PTPN2 (Figures S1A–S1D). We also characterized CD44 and CD62L levels in the 6- to 10-week-old OT-I donor mice we used for our experiments. We found that they were similar, and that the majority of donor cells showed a CD44^low^ CD62L^high^ naive phenotype (Figure S1E). Therefore, we concluded that there is no evidence for a pre-existing condition in peripheral OT-I T cells at the time we used them. Having established that PTPN2 deletion did not alter thymocyte and T cell development, we next determined how PTPN2 deficiency impacts T cell differentiation in pathogenic infection. To this end, we transferred low numbers of PTPN2-deficient *Lck-*Cre;Ptpn2^fl/fl^ OT-I (knockout [KO]) and control Ptpn2^fl/fl^ OT-I CD8 T cells (wild-type [WT]) into CD45.1 congenic C57BL/6 hosts and infected the mice with recombinant *Listeria monocytogenes* (Lm), which was modified to express the ovalbumin-derived SIINFEKL ligand (N4). We observed that the absence of PTPN2 caused a clear shift in the ratio of CD127^−^KLRG1^+^ terminal effector versus CD127^+^KLRG1^−^ memory precursor CD8 T cells upon infection with Lm-N4 (Figures 1A and 1B). This was detectable at 7 days post infection, continued until day 28, and was evident in the spleen and blood of infected animals (Figure 1B). Surprisingly, although WT and PTPN2-deficient OT-I T cells displayed major phenotypic differences, they still responded similarly in magnitude as the ratio of KO over WT T cells remained constant throughout the response (Figures 1C and S1B). Alongside, we noted that PTPN2-deficient CD8 T cells showed prolonged expression of the interleukin (IL)-2 receptor α chain (CD25) when stimulated with Lm-N4 (Figure 1D). This was reflected in an at least 3-fold higher geometric mean fluorescent intensity of CD25 in PTPN2-deficient OT-I CD8 T cells at 4 days post infection (Figure 1D). The enhanced survival of T cells occurred independently of the level of stimulation, as a similar persistence of T cells with a terminal effector phenotype was observed in response to previously described high- and low-affinity OT-I ligands (Figure S2) (Turner et al., 2008; Zehn et al., 2009). Moreover, following low-affinity stimulation, we even observed that absence of PTPN2 temporary shifted the ratio in favor of KO over WT cells (Figure S2), indicating that low-affinity T cell survival is improved, or their transition into the T cell contraction phase is delayed, in the absence of PTPN2. The effects on survival may in part be caused by the increased surface CD25 levels, but the intracellular enhancement of common γ-chain signal transduction in the absence of PTPN2 needs to be considered as a major contributing factor. Of note, PTPN2-deficient OT-I CD8 T cells did not display functional differences on a cell-by-cell basis compared with WT cells. This was demonstrated in an *ex vivo* cytotoxicity assay in which PTPN2-deficient versus WT effector T cells at day 7 post Lm-N4 infection were isolated and incubated with peptide-pulsed splenocytes as target cells in specific ratios (Figure 1E). Altogether, the data demonstrate that the deletion of PTPN2 augments the long-term persistence and expansion capacity of T cells that lack a typical CD127^+^ memory phenotype.

### PTPN2 Deficiency Enables the Re-expansion of KLRG1^+^ T Cell Populations

**Figure 2:**
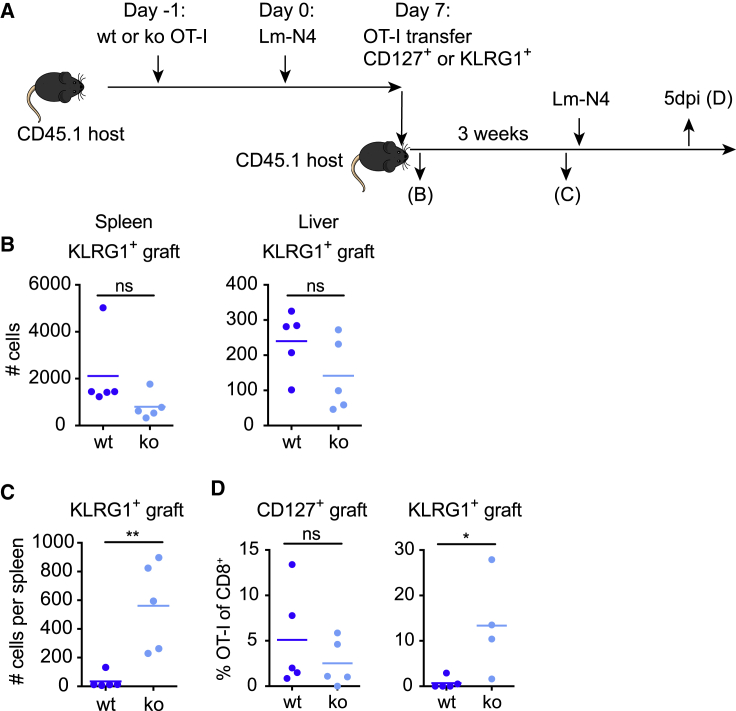
PTPN2-Deficient KLRG1^+^ T Cells Can Undergo Robust Secondary Expansion (A) Experimental outline: CD45-congenic C57BL/6J host mice received 10^4^ WT or PTPN2-deficient T cells and 24 h later 1,000 CFUs of Lm-N4. At 7 days post infection, either KLRG1^+^ effector or CD127^+^ memory precursor PTPN2-deficient or WT OT-I T cells were sorted and adoptively transferred into new hosts. (B–D) Hosts were sacrificed and OT-I numbers were analyzed 24 h (B, KLRG1^+^ grafts only) or 3 weeks after the transfer (C) (shown are data for KLRG1^+^ grafts). (D) In addition, 3 weeks after the transfer, the host mice were infected with 1,000 CFUs Lm-N4 and analyzed 5 days later. Shown are data for KLRG1^+^ and CD127^+^ grafts. The data are representative of three independent experiments with four to five mice each. One dot represents one mouse, and the horizontal line the mean in all plots. Statistical analysis: unpaired t test, ^∗∗^p ≤ 0.001, ^∗^p ≤ 0.01, ^ns^p ≥ 0.05.

As a next step, we thought to determine the functional capacity of the CD127^−^KLRG1^+^ T cells that survive in the absence of PTPN2. To this end, we isolated and transferred CD127^+^KLRG1^−^ and CD127^−^KLRG1^+^ WT and KO OT-I T cells at 7 days post infection with Lm-N4 into naive secondary host mice (Figure 2A). Of note, both types of donor cells showed similar levels of engraftment after the transfer (Figure 2B), although there was a tendency toward slightly lower engraftment of PTPN2-deficient T cells. However, although KLRG1^+^ WT T cells were, as expected, barely detectable 3 weeks later, we found KLRG1^+^ PTPN2-deficient T cells in detectable numbers (Figure 2C). Most significantly, the transferred KLRG1^+^ PTPN2-deficient T cells mounted a robust secondary T cell response following pathogen challenge (Figure 2D). Thus, absence of PTPN2 does not only increase the survival capacity of T cells that lack a typical CD127^+^ memory phenotype, it also enables these cells to undergo secondary expansion. Moreover, taking into consideration the potential to manipulate PTPN2 expression for immunotherapies, it is important to note that we did not find any evidence that eliminating PTPN2 negatively impacts the effector capacity of T cells.

### PTPN2 Deficiency Largely Enhances the Expansion of *Ex Vivo*-Stimulated and Adoptively Transferred T Cells

**Figure 3:**
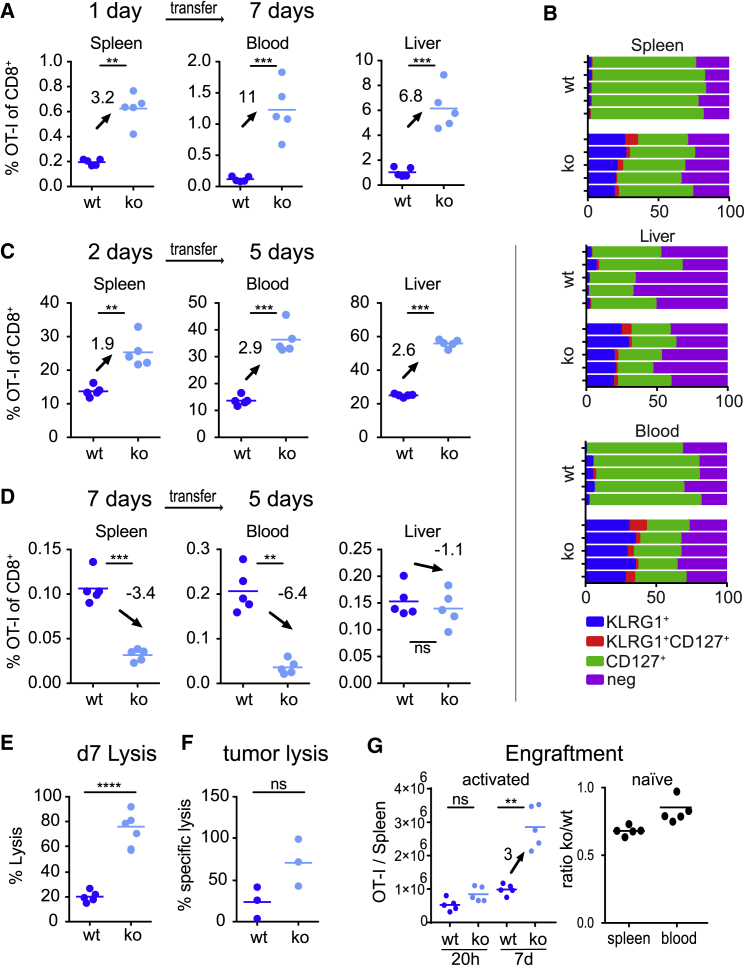
PTPN2 Deficiency Enhances Programmed Expansion of Briefly Stimulated Effector T Cells (A–D) PTPN2-deficient or WT OT-I T cells were activated *in vitro* with SIINFEKL, H-2Kb, and CD80 expressing artificial APCs (MEC.B7.SigOVA) for 1 (A and B), 2 (C), and 7 (D) days. Activated T cells (10^5^) were then transferred into antigen-free CD45-congenic C57BL/6J host mice. (A and B) Frequency of OT-I among total CD8 T cells was determined 7 days post-transfer (A). Bar graphs in (B) show the representative phenotype of five individual mice of the recovered OT-I T cells. (C and D) Frequency of OT-I among total CD8 T cells was determined 5 days post-transfer. (E) Hosts were grafted as in (A), but 7 days after the transfer they received 10^6^ CD45-congenic, carboxyfluorescein succinimidyl ester (CFSE)-labeled target splenocytes that were pulsed with SIINFEKL peptide and 10^6^ CD45-congenic unpulsed control splenocytes which served as a reference population. The plots show the calculated frequency of residual peptide-pulsed target cells at 6 h post-injection in the spleen. (F) Same setup as (E), but 10^5^ Ova- and GFP-expressing RMA cells and antigen-negative mCherry-expressing RMA cells were intraperitoneally (i.p.) injected. The ratio of GFP versus mCherry RMA cells in the peritoneal fluid was determined by flow cytometry 2 days after the transfer. (G) The left plot shows the OT-I T cell numbers recovered per spleen at 20 h post-transfer and at 7 days post-transfer of 10^5^ activated WT versus KO OT-I T cells. The plot to the right shows the ratio of KO/WT OT-I T cells at 6 h post-transfer of 10^6^ naive T cells. The data are representative of five (A and B) or two (C and G) independent experiments with 3–5 mice each. Dots in all panels represent data from a mouse and horizontal lines the mean. Statistical analysis: unpaired t test, ^∗∗∗∗^p ≤ 0.00001, ^∗∗∗^p ≤ 0.0001, ^∗∗^p ≤ 0.001, ^∗^p ≤ 0.01, ^ns^p ≥ 0.05.

The efficacy of adoptive T cell therapies critically depends on the number of engrafted T cells and their *in vivo* expansion magnitude. Adoptive therapies are typically performed with *ex vivo*-activated and -manipulated T cells. Our observations that the deletion of PTPN2 enhances the survival and maintenance of effector T cells prompted us to test how the absence of PTPN2 impacts the adoptive transfer capacity of *ex vivo*-activated T cells. To address this, we used a previously established *in vitro* stimulation system in which T cells undergo the following steps: (1) exposure to antigen and co-stimulation on artificial antigen-presenting cells (APC) for a defined period, (2) separation from the APCs, and (3) then transfer into antigen-free host mice (van Stipdonk et al., 2001). Interestingly, when cells were transferred into antigen-free hosts 1 day after the stimulation, we detected ∼3 times more PTPN2-deficient T cells in the spleen compared with WT cells, ∼11 times more in the blood, and ∼7 times more in the liver (Figure 3A). Phenotypically, the recovered KO OT-I T cells displayed again a bias toward an increased frequency of KLRG1^+^ T cells (Figure 3B). Similar observations with respect to increased T cell expansion and the bias toward KLRG1^+^ effector cells were made for cells stimulated *ex vivo* for 2 days prior to the transfer (Figure 3C). However, this effect was not evident when cells were transferred after 7 days of *ex vivo* culture (Figure 3D), whereupon PTPN2-deficient T cells accumulated in much lower numbers than the WT cells, consistent with a negative effect on engraftment. This reveals the existence of a critical window during which the absence of PTPN2 augments autonomous expansion of activated T cells. We also tested the *in vivo* cytotoxic activity of briefly *ex vivo*-activated and transferred WT and PTPN2-deficient T cells. As expected, we saw higher cytotoxic activity when peptide-loaded and reference target splenocyte populations (Figure 3E) or antigen-negative and antigen-positive tumor cells were injected into host mice that received PTPN2-deficient OT-I cells (Figure 3F). We attribute this higher cytotoxic activity to the increased overall number of PTPN2-deficient T cells but cannot exclude contributions from cell-intrinsic differences in cytotoxic activity as noted previously (Wiede et al., 2014b, 2020). Notably, numbers of PTPN2-deficient and WT OT-I T cells were comparable 20 h after the transfer (Figure 3G). This indicates that better autonomous proliferation and not differences in the transfer efficacy accounts for the increased numbers of PTPN2-deficient T cells. Finally, we evaluated how the *ex vivo*-activated OT-I T cells would respond to cognate antigen stimulation in an infection. We transferred OT-I T cells into mice infected with recombinant Lm-N4 that produce the SIINFEKL ligand for OT-I T cells. In this setup, both WT and PTPN2-deficient OT-I T cells underwent similar expansion (Figure S3C). This excludes that the *ex vivo*-stimulated PTPN2-deficient T cells are in a state of hyperactivation where additional stimulation would lead to activation-induced cell death. In contrast, the ratio shifted on average 160:1 in favor of the PTPN2-deficient OT-I T cells following transfer into WT *Listeria*-infected mice, which do not provide a cognate ligand for OT-I T cells (Lm-WT; Figure S3C). We concluded that the absence of PTPN2 enhances the capacity of T cells to expand autonomously after cessation of TCR signaling. In the past, the capacity for T cells to expand autonomously after being stimulated by antigen and co-stimulation for a brief period has been termed programmed T cell expansion (Bevan and Fink, 2001; van Stipdonk et al., 2001). Therefore, we conclude that the deletion of PTPN2 enhances programmed T cell expansion and increases the size of the T cell population that forms after transferring previously activated T cells into host mice.

### Deletion of PTPN2 Alters Cytokine Signal Transduction in Recently Activated T Cells

**Figure 4:**
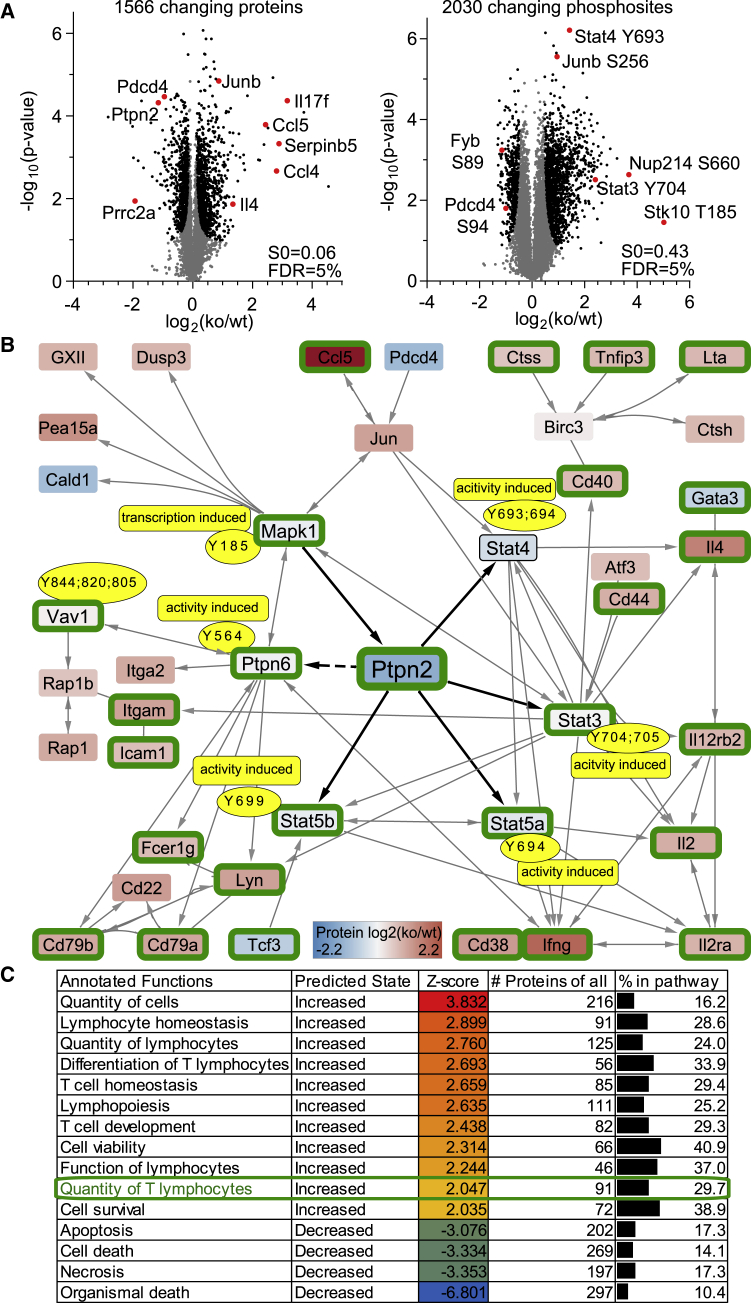
Proteome Analysis of Briefly Stimulated WT and PTPN2-Deficient T Cells (A) Volcano plots showing quantitative changes of protein and phosphosite levels of PTPN2-deficient versus WT OT-I T cells that were activated for 30 h *in vitro* with anti-CD3/anti-CD28-coupled beads. Significant hits (black) compared with non-significant hits (gray) were determined via a constant S0, which was calculated in R (version 3.4.1, function “samr”) and further corrected for multiple testing by applying a permutation-based 5% false discovery rate (FDR) calculation. (B) To extract signaling cascades that are markedly deregulated in the absence of PTPN2, we used the *de novo* network enrichment tool KeyPathwayMiner. This used the interactome of the murine STRING network (v.11) and known interactions of differentially phosphorylated tyrosine phosphosites curated in the IPA database to extract the key regulatory network of these datasets: (1) differentially phosphorylated tyrosine phosphoproteins (absolute log2 KO/WT fold change > 0.5 and p < 0.05), and (2) differential protein expression (absolute log2 fold change > 0.5 and p < 0.05). Depicted is the largest subnetwork that has been extracted by KeyPathwayMiner. This has been overlaid with protein expression data and function of differentially phosphorylated tyrosine residues. Highlighted with a green border are the proteins that are associated with an increased quantity of T lymphocytes as designated by the IPA analysis. (C) Analysis of changes in protein expression and phosphorylation status via the IPA software. Probability index (*Z* score) that links PTPN2 KO versus WT datasets to specific signatures.

The greatly improved expansion capacity of briefly activated T cells in the absence of PTPN2 prompted us to use proteomics to assess the mechanisms by which elimination of PTPN2 can enhance T cell survival and programmed expansion. We analyzed 30-h *ex vivo*-activated WT or PTPN2-deficient OT-I T cells and detected 1,566 differentially expressed proteins and 2,030 phosphorylated sites with differential abundance (Figure 4A). Of these, 24 were phospho-tyrosine sites. This low number is not unexpected given that they are generally underrepresented compared with serine and threonine phosphorylations (Huttlin et al., 2010; Lundby et al., 2012). The detected tyrosine residues include many known but also previously unrecognized sites (Table S1). To explore connections between the alterations in protein and phosphorylation levels, we took advantage of the network enrichment tool KeyPathwayMiner (List et al., 2016; Pauling et al., 2014), which extracts *de novo* pathways, i.e., enriched sub-networks that are part of a protein-protein interaction network (for details, see the STAR Methods). Known protein-protein interactions were obtained from the murine STRING network (v.11, Szklarczyk et al., 2019) and the database embedded in the Ingenuity pathway analysis tool (IPA; v.46901286; QIAGEN) (Krämer et al., 2014). Figure 4 shows the largest sub-network reported by KeyPathwayMiner. Hub nodes in this network are STAT3, STAT4, and STAT5, which are hyperphosphorylated and in a transcriptionally active state in the absence of PTPN2. These hubs are linked to a larger number of differentially expressed proteins. These include increased levels of proliferation-associated proteins, such as IL-2, IL-2rα, interferon (IFN)-γ, IL-4, CD44, and several others (Figure 4B). Subsequently, we analyzed the changes directly via IPA and determined the probability index (*Z* score) that links PTPN2 deficiency-related proteome alterations to specific functions and disease-related signatures. The absence of PTPN2 was shown to correlate with enhanced proliferation-related signatures and decreased apoptosis activity scores (Figure 4C). Altogether, our data suggest that the absence of PTPN2 predominantly increases the responsiveness to common γ-chain cytokines and consequently augments co-stimulation-independent expansion and survival of recently activated T cells.

### PTPN2 Deficiency Increases IL-2- and IL-15-Induced Signal Transduction in T Cells during Programmed Expansion

**Figure 5:**
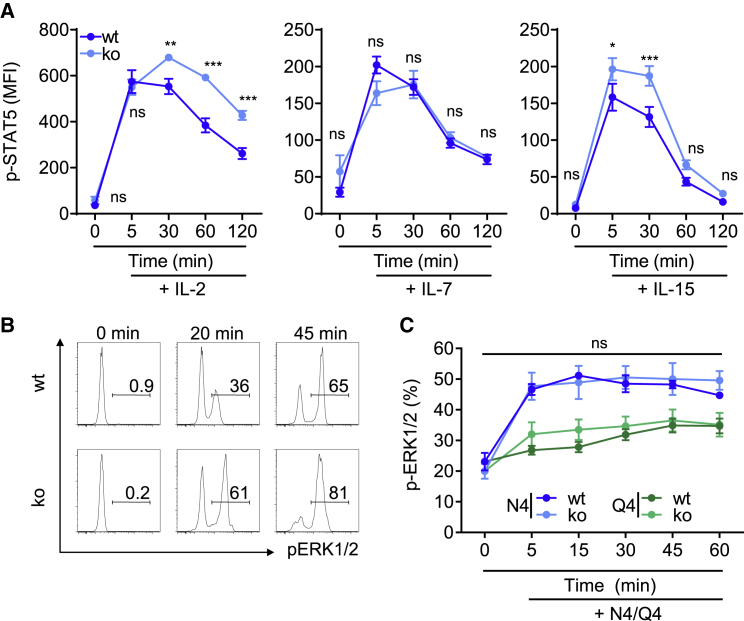
PTPN2 Deficiency Enhances Common γ-Chain-Mediated Cytokine Signaling (A) PTPN2-deficient or WT OT-I T cells were activated *in vitro* with SIINFEKL, H-2Kb, and CD80 expressing artificial APCs (MEC.B7.SigOVA) for 36 h, rested for 1 h, and then stimulated with IL-2 (5 ng/mL), IL-7 (10 ng/mL), or IL-15 (20 ng/mL) for the indicated times. Cells were then intracellularly stained with anti-(pY694)-STAT5 (p-STAT5), and the MFI for p-STAT5 was determined by flow cytometry. (B) A total of 10^5^ naive CD8^+^ T cells from WT and PTPN2-deficient mice were incubated with 3 × 10^5^ bone marrow-derived dendritic cells pulsed with SIINFEKL (0.1 mg/mL) for the indicated time points. Cells were intracellularly stained with anti-p-ERK1/2, and the percentage of p-ERK1/2 was determined by flow cytometry. (C) The activated OT-I T cells were serum starved for 8 h and stimulated with SIINFEKL (0.1 mg/mL) or SIIQFEKL (0.1 mg/mL) for the indicated time points. Cells were intracellularly stained with fluorochrome-conjugated anti-(pT202/pY204)-ERK1/2 (p-ERK1/2), and the percentage of p-ERK1/2 was determined by flow cytometry. The data are representative of two independent experiments with 3–5 mice each. Dots in all panels represent the mean, and error bars the SEM. Statistical analysis: unpaired t test, ^∗∗∗^p ≤ 0.0001, ^∗∗^p ≤ 0.001, ^∗^p ≤ 0.01, ^ns^p ≥ 0.05.

Our proteomic data indicate a higher capacity of recently activated PTPN2-deficient T cells to respond to common γ-chain cytokines, a notion that is well in line with prior reports of the function of PTPN2 (Wiede et al., 2011; Zikherman and Weiss, 2011). To experimentally verify this, we activated OT-I T cells for 36 h, starved them for 8 h in serum-free medium, and stimulated them with IL-2, IL-7, or IL-15 for a defined period. We observed that PTPN2-deficient OT-I T cells show increased IL-2- and IL-15-induced signaling, which was reflected in enhanced phosphorylation levels of STAT5-Y694 (Figure 5A), confirming our proteomic data. Subsequently, we explored whether activated PTPN2-deficient T cells also show an altered TCR signal transduction capacity as occurs in naive cells (Wiede et al., 2011, 2014b, 2017). To this end, we stimulated naive versus activated WT and PTPN2-deficient OT-I T cells with SIINFEKL pulsed bone marrow-derived dendritic cells (BMDCs) and monitored for the TCR-induced extracellular signal-regulated kinases 1 and 2 (ERK1/2) signaling. In naive OT-I T cells, PTPN2 deficiency increased TCR-induced ERK1/2 phosphorylation (p-ERK1/2) (Figure 5B). By contrast, PTPN2-deficient OT-I T cells that were previously activated and underwent programmed expansion did not display increased p-ERK1/2 after re-stimulation with SIINFEKL or with lower-avidity SIIQFEKL peptide when compared with WT cells (Figure 5C). This demonstrates that PTPN2 deficiency enhances the capacity of recently activated T cells to respond to IL-2 and IL-15, but not to TCR ligation.

### PTPN2 Deficiency Enhances IL-2 Sensitivity and Survival Capacity of Recently Activated T Cells

**Figure 6:**
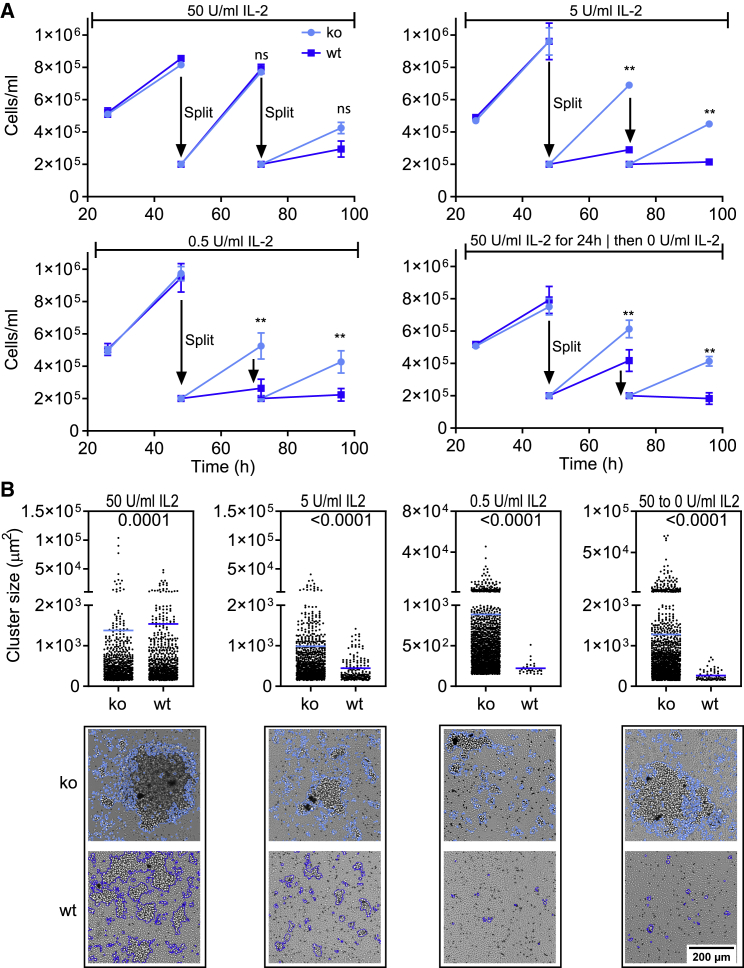
PTPN2 Deficiency Increases IL-2 Sensitivity of Recently Activated T Cells (A) A total of 5 × 10^5^ PTPN2-deficient or WT OT-I T cells were activated with anti-CD3/anti-CD28-coupled beads and stimulated with the indicated concentrations of IL-2. Activated cells were split every 24 h, and 2 × 10^5^ cells were transferred into new wells. (B) The dot plots show the number and size of clusters at 72 h after activation, and each dot represents one cluster of a size >200 μm. The images show a representative example of cluster determination via ilastik (v.1.3.2) as indicated by the blue outline. The number of clusters was counted via ImageJ (v.1.5). Shown data are three replicates that are representative of three independent experiments. Statistical analysis: (A) unpaired t test, ^∗∗^p ≤ 0.001, ^ns^p ≥ 0.05. (B) Nonparametric Mann-Whitney test with indicated p values.

Our signaling data showed increased responses of recently activated PTPN2-deficient T cells to common γ-chain cytokines. Nonetheless, it remained unclear whether this had functional consequences on T cell proliferation. To address this, we activated OT-I T cells using anti-CD3/anti-CD28-coated beads and exposed them to titrated doses of IL-2. To correct for the increased cellular expansion, we split cells every 24 h and re-seeded them at a similar density in fresh wells and supplemented with 50% conditioned medium and 50% fresh medium and the respective amounts of IL-2. We observed that PTPN2-deficient cells showed better survival following the provision of limited IL-2 levels compared with WT cells (Figure 6A). As a hallmark of robust and functional proliferation, we also assessed the ability of cells to form proliferative clusters. As determined via microscopy and automated cluster determination and counting, we observed that PTPN2-deficient cells showed higher cluster-forming capacity following exposure to low IL-2 levels (Figure 6B). Our data demonstrate that recently activated PTPN2-deficient T cells go through an interval of heightened IL-2 sensitivity, and that much lower IL-2 concentrations are sufficient to maintain PTPN2-deficient T cells in a proliferating state. Altogether, our data strongly suggest that this improved IL-2 sensitivity results in the increased expansion of PTPN2-deficient T cells.

## Discussion

PTPN2 has received considerable attention because reductions in its expression correlate with the development of autoimmune diseases (Espino-Paisan et al., 2011; Smyth et al., 2008; Todd et al., 2007; Zikherman and Weiss, 2011). Moreover, its known role in negatively regulating TCR sensitivity through dephosphorylation of SRC family kinases and in attenuating JAK-STAT-mediated cytokine signaling has made it an attractive target for immunotherapy (LaFleur et al., 2019; Wiede et al., 2020). However, how complete absence of PTPN2 impacts basic aspects of CD8 T cell effector and memory differentiation and their protective capacity has remained unknown. Our data revealed that the absence of PTPN2: (1) augments or prolongs the survival of short-lived effector T cell populations, and (2) alters the developmental plasticity of transferred effector T cell populations, and also that (3) targeting PTPN2 in recently activated T cells promises to have significant therapeutic benefits.

KLRG1-specific T cells are known to arise in large numbers in many types of acute infection (Joshi et al., 2007; Kaech et al., 2003; Wherry et al., 2003). They are thought to predominantly mark cells that have turned toward the final stage of differentiation and lost the ability to form memory T cells. This was concluded given the limited capacity of transferred KLRG-1^+^ T cells to undergo secondary expansion (Sallusto et al., 2000; Sarkar et al., 2008; Schluns et al., 2000; Weinreich et al., 2009). Although the concept of exclusive terminal differentiation of KLRG1^+^ T cells was recently challenged (Herndler-Brandstetter et al., 2018), the notion that these cells have a limited capacity to become engrafted into new hosts is well established. Much to our surprise, we noted that transferred PTPN2-deficient CD127^−^KLRG1^+^ T cells showed superior secondary expansion capacity. These results indicate that absence of PTPN2 enhances the survival of KLGR1^+^ cells and enables responses of these cells that resemble the function of conventional memory T cells.

Most strikingly, our data underline the potential benefits that may result following manipulations of PTPN2 expression in therapeutically relevant adoptive T cell transfer settings. We demonstrate that the absence of PTPN2 enables cells to undergo better programmed expansion following a limited period of stimulation. Notably, we saw that the engraftments of WT and KO cells were initially similar. Only subsequently did the PTPN2-deficient cells expand better, which is why we consider that the absence of PTPN2 augments programmed T cell expansion. This ability could be clinically beneficial because it remains a major challenge to engraft patients with sufficient numbers of functional T cells. Increasing the efficacy of T cell transfers by 3- to 10-fold would constitute a major advantage. However, the limited time window of 24–48 h post-activation during which we observed this beneficial effect may somewhat limit potential. Nonetheless, we envision that targeting PTPN2 genetically, or with inhibitors, in briefly activated T cells that were transduced with chimeric antigen receptors (CAR T cells) could be very beneficial as reported recently (Wiede et al., 2020). Here, cells could be activated for 24–36 h, transduced with a CAR, and in parallel the expression of PTPN2 could be manipulated, and shortly thereafter the cells could be transferred into patients. According to our mouse studies, this should result in a significant increase in transfer efficacy, improving the overall therapeutic outcome.

All of our observations suggest that the absence of PTPN2 enhances the survival of activated T cells. Given the previously identified increases in common γ-chain-mediated cytokine signaling, we addressed the possible contribution to the improved transfer capacity using a proteomics approach. Using the *de novo* network enrichment tool KeyPathwayMiner, we observed strong correlations between alterations in total protein levels and increased phosphorylation status of known and newly identified PTPN2 interaction partners. This included heightened phosphorylation status and activity in STAT3, STAT4, and STAT5, and enhanced expression of proliferation-associated proteins, such as IL-2, IL-2rα, IFN-γ, IL-4, CD44, CCL5, JUN, and several others. Altogether, these observations imply that PTPN2-deficient T cells are less dependent on receiving pro-survival signals than WT cells or may enable T cells to still effectively respond to limiting cytokine sources, both of which could explain improved transfer efficacy. Functionally, we were able to demonstrate that these changes render PTPN2-deficient T cells much less dependent on IL-2 signaling *in vitro*. Likewise, absence of PTPN2 might render CD8 T cells more responsive to IL-2 that is pharmacologically applied in combinatorial therapies. Furthermore, we observed that the absence of PTPN2 increased the capacity to form clusters in *in vitro* cultures, which is considered a hallmark of robust T cell proliferation. This augmented cluster formation also matches the observed changes in PTPN2-deficient T cell expression of integrin alpha-2 (ITGA2) and integrin alpha-M (ITGAM), as well as its corresponding binding partner intercellular adhesion molecule 1 (ICAM1) (Figure 4). Moreover, our proteomic network analysis also suggests potential novel interactors or downstream affected molecules of PTPN2. This includes PTPN6 (SHP-1), which has been extensively studied as a negative regulator of TCR signaling (Carter et al., 1999; Johnson et al., 2013). Similarly, the decreased expression of the tumor suppressor and apoptosis-inducing factor PDCD4 might play a role in the increased survival of PTPN2 KO T cells (Shibahara et al., 1995; Yang et al., 2003). It has recently been described that cytotoxic T cells lacking PDCD4 expression show increased expression of effector molecules and display superior tumor control (Lingel et al., 2017).

Our data also complement and extend the recently described role of PTPN2 in the differentiation of T cells in chronic infections (LaFleur et al., 2019). This study demonstrates that TIM3^+^ terminal effector T cells proliferate more strongly and can in enhanced numbers contribute to viral control, which is biologically comparable with our conclusion of prolonged effector T cell survival in the absence of PTPN2. Likewise, PTPN2-deficient T cells were also able to markedly improve tumor therapy in syngeneic tumor models (LaFleur et al., 2019; Wiede et al., 2020).

Overall, our work reveals PTPN2 as a critical factor for effector and recently activated T cell populations. Notably, we did not observe any adverse effects of eliminating PTPN2, because neither effector capacity nor the ability to form memory or to undergo secondary expansion was compromised. In conclusion, our data further support the development of PTPN2 inhibitors for adoptive T cell-based therapies, because the deletion or inhibition of PTPN2 would significantly improve the survival and expansion of transferred T cells and confer improved immunity through strengthened T cell numbers.

## STAR★Methods

### Key Resources Table

**Table:** 

REAGENT or RESOURCE	SOURCE	IDENTIFIER
**Antibodies**		

CD44 (IM7)	Biolegend	103012; RRID: AB_312963
CD25 (PC61.5)	eBioscience	25-0251-82; RRID:AB_469608
CD8α (53-6.7)	Biolegend	100714; RRID: AB_312753
CD45.1 (A20)	eBioscience	48-0453-82; RRID: AB_1272189
CD45.2 (104)	eBioscience	17-0454-82; RRID: AB_469400
CD127 (A7R34)	eBioscience	17-1271-82; RRID: AB_469435
KLRG1 (2F1)	eBioscience	48-5893-82; RRID: AB_10852843
Vα2 TCR (B20.1)	eBioscience	17-5812; RRID: AB_1659733
FITC (NAWESLEE)	eBioscience	12-7691-82; RRID: AB_2572663
PE (eBioPE-DLF)	eBioscience	13-4120-82; RRID: AB_529611
Phospho-Stat5 (Y694)	Cell signaling technologies	#9351; RRID: AB_2315225
Phospho-p44/42 MAPK (Erk1/2) (Thr202/Tyr204)	Cell signaling technologies	#9101; RRID: AB_331646
Anti-rabbit IgG DyLight 649	JacksonImmunoResearch	211-492-171; RRID: AB_2339164
CD69 (H1.2F3)	eBioscience	12-0691-82; RRID: AB_465732
CD90.1 (OX-7)	Biolegend	202503; RRID: AB_314014
CD24 (M1/69)	BDBiosciences	563060; RRID: AB_2737981
TCR Vβ 5.1/5.2 (MR9-4)	eBioscience	46-5796-82; RRID: AB_10853817
CD62L (MEL-14)	eBioscience	25-0621-82; RRID: AB_469633

**Bacterial and Virus Strains**		

*Vesicular stomatitis virus* (VSV), VSV-N4, VSV-V4	(Kim et al., 1998)	Ref-SKU: 014V-02165
*Listeria monocytogenes* (Lm), Lm-N4, Lm-V4, Lm-Q4, Lm-wt	(Zehn et al., 2009)	N/A

**Chemicals, Peptides, and Recombinant Proteins**		

Recombinant murine IL-2	Peprotech	5696121
Dynabeads Mouse T-Activator antiCD3/antiCD28 beads	Thermo Fisher	11456D
SIINFEKL	JPT Peptide Technology and EMC microcollections	BAP-201
SIIQFEKL	JPT Peptide Technology and EMC microcollections	N/A
Recombinant murine IL-7	Peprotech	217-17
Recombinant murine IL-15	Peprotech	210-15
Critical Commercial Assays		
TMTsixplex reagents	Thermo Fisher	90066
CD8^+^ T cell-enrichment kit	Miltenyi Biotech	130-096-495

**Deposited Data**		

Proteomics data have been deposited with the PRIDE partner repository (Vizcaíno et al., 2013)	ProteomeXchange Consortium	PRIDE: PXD013122

**Experimental Models: Cell Lines**		

RMA cells	M.J. Bevan, University of Washington, batch of 9.11.1989	RRID:CVCL_J385
MEC.B7.SigOVA (express SIINFEKL, H-2Kb and CD80)	(van Stipdonk et al., 2001)	N/A

**Experimental Models: Organisms/Strains**		

OT-I;*Lck-*Cre;*Ptpn2*^*fl/fl*^ (KO)	(Wiede et al., 2011)	N/A
OT-I;*Ptpn2*^*fl/fl*^ (WT)	(Wiede et al., 2011)	N/A
C57BL/6J host mice	(Schluns et al., 2002)	MGI: J:109854

**Software and Algorithms**		

MaxQuant (v1.6.0.16)	(Cox and Mann, 2008)	https://www.maxquant.org/
ImageJ (v1.5)	(Schneider et al., 2012)	https://imagej.nih.gov/ij/
KeyPathwayMiner	(List et al., 2016)	https://keypathwayminer.compbio.sdu.dk/keypathwayminer/
IPA version 46901286	(Krämer et al., 2014), QIAGEN Inc	N/A
ilastik object classification and segmentation software (v. 1.3.2, 53)	(Sommer et al., 2011)	https://www.ilastik.org/index.html
Prism 8.0	Graphpad Software	N/A
FlowJo (v10.1)	Treestar	https://www.flowjo.com/
Graphical abstract created with BioRender.com	Biorender	https://biorender.com/

### Resource Availability

#### Lead Contact

Further information and requests for resources and reagents should be directed to and will be fulfilled by the Lead Contact, Dietmar Zehn (dietmar.zehn@tum.de).

#### Materials Availability

This study did not generate new unique reagents.

#### Data and Code Availability

The MS proteomics data and unprocessed MaxQuant search results have been deposited with the ProteomeXchange Consortium (http://www.proteomexchange.org/) via the PRIDE partner repository (Vizcaíno et al., 2013) with the dataset identifier PRIDE: PXD013122.

### Experimental Model and Subject Details

#### Mice and adoptive T cell transfers

OT-I;*Lck-*Cre;*Ptpn2*^*fl/fl*^ (KO) and OT-I;*Ptpn2*^*fl/fl*^ (WT) mice have been described previously (Wiede et al., 2011). Donor cells were adoptively transferred into CD45.1 congenic C57BL/6J host mice (Schluns et al., 2002) for proliferation tests and infection experiments. In some experiments, WT (CD45.1/2) and PTPN2-deficient OT-I T cells (CD45.2/2) were co-transferred into CD45.1 congenic host mice. All mouse breeding was performed in specific pathogen-free facilities and experimental procedures were performed in six to twelve week old female or male mice in modified specific pathogen-free animal facilities. Initial experiments were performed at the University of Lausanne in Switzerland and were in compliance with institutional and governmental regulations of the Swiss Canton Vaud. Later, experiments were performed at the Technical University of Munich in Germany and in accordance with institutional and governmental regulations of the Regierung von Oberbayern. Experimental groups were non-blinded, animals were randomly assigned to experimental groups and no specific method was used to calculate sample sizes.

#### Infections

Previously described recombinant versions of *Vesicular stomatitis virus* (Indiana strain) that express either the normal high avidity OT-I epitope SIINFEKL (N4) Kim et al., 1998 or the low avidity OT-I altered peptide ligand SIIVFEKL (V4) Zehn, 2010 were grown and titrated on BHK cells. Mice were infected intravenously (i.v.) with 2 × 10^6^ PFU. Wild-type *Listeria monocytogenes* and recombinant *Listeria monocytogenes* strains expressing wt ovalbumin or ovalbumin containing K^b^/Ova-derived APL were also previously described (Zehn et al., 2009). All *Listeria* were grown in brain heart infusion broth (Oxoid, Thermo Fisher) to mid-log phase and bacterial numbers were determined by measuring the OD_600_. Mice were i.v.-infected with 1000 colony-forming units (CFU).

#### *In vitro* T cell activation and proliferation assays

Isolated naive PTPN2-deficient and WT CD8 T cells were activated with Dynabeads Mouse T-Activator antiCD3/antiCD28 beads (Thermo Fisher), in accordance with the manufacturer’s instructions, and varying concentrations of recombinant IL-2 in RPMI medium supplemented with 10% heat inactivated FCS, 5 mM HEPES (Invitrogen), 50 μM β-Mercaptoethanol (Invitrogen), and 100 U/ml of Penicillin and Streptomycin (Amimed). Cell numbers were determined in Neubauer counting chambers as well as by flow cytometry. For determination of proliferative clusters the ilastik object classification and segmentation software (v. 1.3.2, 53) was used and clusters were counted automatically using the ilastik segmentation classification criteria for proliferative clusters of a size over 200 μm in ImageJ (v1.5). For testing T cell programing and autonomous expansion, PTPN2-deficient and WT OT-I T cells were isolated and activated in accordance with a previously established *in vitro* stimulation system. Briefly, OT-I T cells were co-cultured with IFN-γ primed MEC.B7.SigOVA adherent fibroblasts engineered to express SIINFEKL, H-2K^b^ and CD80 (van Stipdonk et al., 2001). Cells were cultured in IL-2 supplemented medium as indicated above. After 1, 2 or 7 days of *in vitro* culture, antigen, and co-stimulation was terminated via separation from the APCs. Then, 10^5^ activated T cells were transferred into antigen-free CD45-congenic C57BL/6J host mice and organs were harvested 5-7 days later.

### Method Details

#### Donor cell preparation, purification, and labeling

Single-cell suspensions were obtained by mincing spleens with a scalpel and then by mashing them through a 100 μm nylon cell strainer (BD Falcon). Red blood cells were lysed with hypotonic ammonium-chloride-potassium (ACK) lysis buffer. The mouse CD8^+^ T cell-enrichment kit (Miltenyi Biotech) was used for CD8 T cell-isolation. Memory OT-I T cells were generated by transferring low numbers of naive OT-I T cells into CD45.1 congenic host mice and by infecting the hosts with 1000 CFU of Lm-N4. Memory cells were re-isolated via staining and positive selection of live cells in 2% FCS RPMI medium (Sigma Aldrich). First, cells were stained with fluorescein isothiocyanate (FITC)-KLRG1, or phycoerythrin (PE)-CD127, and biotin-conjugated CD45.2 antibodies, and then enriched via MACS separation using anti-biotin MicroBeads (Miltenyi Biotech), in accordance with the manufacturer’s instructions. Afterward, cells were sorted for KLRG1^+^ or CD127^+^ populations using a FACS Aria Fusion instrument (BD) and then transferred into new CD45.1/1-congenic C57BL/6J host mice.

#### Surface protein antibody staining and flow cytometry analysis of mouse cells

Staining of extracellular proteins was performed with the aforementioned antibodies for 30 min at 4 °C in FACS buffer: PBS (Invitrogen) supplemented with 2% FCS (Sigma Aldrich) and 0.01% azide (Sigma-Aldrich). Then, cells were fixed for 10 min at room temperature in PBS supplemented with 4% formaldehyde, 2% glucose and 0.03% azide. After resuspension in FACS buffer, flow cytometry analysis was performed on an LSR-Fortessa or LSR-II flow cytometer (BD). All data were analyzed using FlowJo (v9.1 and v10.3, TreeStar).

#### Detection of cytokine signaling

OT-I T cells were co-cultured with IFN-γ primed MEC.B7.SigOVA for 36 h and then rested in RPMI supplemented with 1% FCS for 1 h. Afterward, they were stimulated with mouse recombinant IL-2 (5 ng/ml), IL-7 (10 ng/ml) or IL-15 (20 ng/ml) (Peprotech). Then, cells were fixed in Cytofix Fixation Buffer (BD Biosciences) for 15 min at 37°C, washed with D-PBS and permeabilized in methanol/acetone (50:50) overnight at −20°C. The next day, the cells were stained with Phospho-Stat5 (Y694) (D47E7 XP® rabbit, Cell Signaling Technology, 1:400) in D-PBS supplemented with 5% FCS for 1 h at room temperature. Secondary antibodies against rabbit IgG (H+L) F(ab’)2 fragment coupled to DyLight 649 (Jackson ImmunoResearch; 1:800) were used for detection of p(Y694)-STAT5.

#### Generation of bone marrow derived dendritic cells (BMDCs) to assess TCR signaling

Bone marrow was isolated from the tibia and femur of one 8 week old C57BL/6 mouse and erythrocytes were removed with Red Blood Lysing Buffer (Sigma-Aldrich). Then, 6 × 10^6^ isolated bone marrow cells were cultured in a 6 well dish in RPMI medium supplemented with 10% heat inactivated FCS, 5 mM HEPES (Invitrogen), 50 μM β-Mercaptoethanol (Sigma), 100 U/ml of Penicillin and Streptomycin (Invitrogen) and GM-CSF (10 ng/ml) (Peprotech) for 5 days at which point BMDCs were harvested and processed for T cell signaling.

#### Detection of TCR signaling

Cells were serum-starved for 8 h and stimulated with SIINFEKL (0.1 μg/ml) or SIIQFEKL (0.1 μg/ml) (JPT Peptide Technology) pulsed BMDCs for defined periods and then fixed in Cytofix Fixation Buffer (BD Biosciences) for 15 min at 37°C. Afterward, cell were permeabilized with methanol/acetone (50:50) overnight at −20°C and then intracellularly stained with Phosho-p44/42 MAPK (Erk1/2) (Thr202/Tyr204) (Rabbit, Cell Signaling Technology, 1:400) in D-PBS supplemented with 5% FCS for 1 h at room temperature. Secondary antibodies against rabbit IgG (H+L) F(ab’)2 fragment coupled to DyLight 649 (JacksonImmunoResearch; 1:800) were used for detection.

#### Cytotoxicity assays

*In vitro* killing assays were performed with WT and PTPN2 KO OT-I isolated from day 7 Lm-N4 infected hosts. 2 × 10^4^ of the isolated KO or WT cells were *ex vivo* labeled with CSFE and incubated with DAPI (Peprotech) labeled, SIIQFEKL pulsed (EMC microcollections) splenocytes or unpulsed splenocytes as a control for 18 h in RPMI. Specific lysis of peptide-pulsed target cells was determined via flow cytometry. *In vivo* cytotoxicity assays were performed 7 days post transfer of 1 × 10^5^ 24-hour *ex vivo* activated ko or wt OT-I T cells. For Figure 3E 4 × 10^6^ CFSE labeled splenocytes pulsed with SIINFEKL peptide (EMC microcollections) or unpulsed control splenocytes were co-injected i.v. as target cells. Specific lysis of pulsed splenocytes was measured 6h post-injection in the spleen. For Figure 3F 1 × 10^5^ Ovalbumin and eGFP-expressing RMA (RMA-eGFP-Ova) or antigen negative mCherry-expressing RMA cells were co-injected intraperitoneal (i.p.). Cells were harvested two days later by peritoneal lavage and specific lysis of RMA-eGFP-Ova cells was determined by flow cytometry.

#### Sample preparation for mass spectrometry

OT-I CD8 T cells of 3 mice each of PTPN2-deficient and wt mice were isolated and activated for 30 h with Dynabeads Mouse T-Activator antiCD3/antiCD28 beads (Thermo Fisher) in accordance with the manufacturer’s instructions and 50 U/ml of recombinant IL-2 in RPMI media supplemented with 10% heat inactivated FCS. Cellular proteins were extracted using urea lysis buffer (8 M urea, 40 mM Tris-HCl (pH 7.6), protease inhibitor cocktail, phosphatase inhibitor cocktail), and lysates were cleared by centrifugation (15 min, 20.000 × g, 4°C). Protein concentration of the supernatant was determined by the Bradford method (Coomassie Protein Assay Kit, Thermo Fisher Scientific), and 165 μg protein per sample were reduced (10 mM dithiothreitol, 30°C, 30 min) and alkylated (50 mM chloroacetamide, room temperature, 30min, in the dark). Lysates were diluted to 1.6 M urea using 40mM Tris-HCl (pH 7.6), and protein digestion was performed overnight (37°C, 600 rpm) using trypsin (Promega, 1:50 enzyme-to-substrate ratio). Acidified digests were desalted using 50 mg tC18, reversed-phase (RP) solid-phase extraction cartridges (Waters Corp.; wash solvent: 0.1% formic acid (FA); elution solvent: 0.1% FA in 50% acetonitrile (ACN)) and vacuum dried. TMT labeling, phosphopeptide enrichment, high pH reversed phase (RP) tip fractionation of the phosphoproteome, and Trinity P1 fractionation of the full proteome were performed as described previously (Ruprecht et al., 2017; Yu et al., 2017; Zecha et al., 2019). In brief, peptides from triplicates of PTPN2 ko and wt were reconstituted in 20 μL of 50 mM HEPES (pH 8.5) and labeled for 1h at 25°C and 500 rpm using 100 μg TMTsixplex reagents (Thermo Fisher) in 5 μL anhydrous acetonitrile (ACN) per sample. The labeling reaction was stopped by adding 2 μL of 5% hydroxylamine. The acidified and pooled sample was frozen at −80°C, dried down by vacuum centrifugation, and desalted using 50 mg tC18, RP solid-phase extraction cartridges (wash solvent: 0.07% trifluoroacetic acid (TFA); elution solvent: 0.07% TFA in 50% ACN). The desalted peptide solution was adjusted to a final concentration of 30% ACN and loaded onto a ProPac IMAC-10 column (4 × 50 mm, Thermo Fisher Scientific) charged with FeCl_3_ and connected to an Aekta HPLC system. The flow through (non-phosphopeptides) was collected, and bound phosphopeptides were subsequently eluted and collected applying a 6.5min gradient from 0% to 26% IMAC elution solvent (0.315% NH_4_OH) in IMAC loading solvent (30% ACN, 0.07% TFA). Both fractions were vacuum dried. The phosphopeptide fraction was desalted (wash solvent: 0.1% FA) using self-packed StageTips (five disks, Ø 1.5 mm, C18 material, 3 M Empore™), and bound peptides were then washed with 25 mM ammonium formate (pH 10) and sequentially eluted using 40μL of ammonium formate (pH 10) containing increasing ACN concentrations (5%, 7.5%, 10%, 12.5%, 15%, 17.5%, and 50% ACN). The wash flow through was combined with the 17.5% ACN eluate and the 5% ACN fraction with the 50% ACN fraction, resulting in a total of six fractions which were vacuum dried. Non-phosphopeptides were reconstituted in 10 mM ammonium acetate and 100 μg were loaded onto an Acclaim Trinity P1 column (2.1 × 150 mm, 3 μm, Thermo Fisher Scientific) and separated using increasing concentrations of 10mM ammonium acetate in 95% ACN (up to 84%). Thirty-two 1 min fractions were collected and vacuum dried.

#### LC-MS/MS measurements

Nano-flow LC-MS/MS measurements were performed using an Ultimate 3000 RSLCnano system coupled to a Fusion Lumos Tribrid mass spectrometer (Thermo Fisher Scientific). High pH RP fractions containing phosphopeptides were dissolved in 0.1% FA, injected twice and separated at 300 nL/min on an analytical column (75 μm x 45 cm, packed in-house with 3 μm C18 resin; Reprosil Gold, Dr. Maisch GmbH) using an 80min linear gradient from 4% to 32% LC solvent B (0.1% FA, 5% DMSO in ACN) in LC solvent A (0.1% FA in 5% DMSO). MS1 spectra were recorded in the orbitrap at a resolution of 60K using an automatic gain control (AGC) target value of 4e5 charges and a maximum injection time (maxIT) of 20 ms. For the first injection, MS2 spectra for peptide identification were obtained in the orbitrap at 15K resolution via sequential isolation of up to 10 precursors (0.7 m/z isolation window, 5e4 AGC target, 22ms maxIT, 90 s dynamic exclusion) and fragmentation via collisional induced dissociation (CID) enabling multistage activation (35% collision energy, 0.25 activation Q, neutral loss mass of 97.9763). For the second injection, peptides were fragmented via higher energy collisional dissociation (HCD) using a normalized collision energy (NCE) of 33%. Then, for each peptide precursor, an additional MS3 spectrum for TMT quantification was obtained in the orbitrap at 15K resolution (1e5 AGC target, 50 ms maxIT, 100-1.000 m/z scan range). For this, the precursor was again fragmented as for MS2 analysis, followed by synchronous selection of the 10 most intense peptide fragments and further fragmentation via HCD using a NCE of 55%. Trinity P1 fractions were analyzed as single injections as described above with following modifications: Peptides were separated at using a 50 min linear gradient from 8% to 34% LC solvent B in LC solvent A. MS2 spectra were obtained after fragmentation via CID without multistage activation (35% collision energy, 0.25 activation Q, 60 s dynamic exclusion).

#### Analysis of mass spectrometry data

Peptide and protein identification and quantification were performed using MaxQuant (v1.6.0.16) (Cox and Mann, 2008). Tandem mass spectra were searched against the mouse reference database (UP000000589, downloaded on 03.08.2018) supplemented with common contaminants. All search parameters were left as default apart from the following: High pH RP and Trinity P1 fractions were separated into two groups and ”6plex TMT” was specified as a label within the reporter ion MS3 experiment type. Phosphopeptide samples were defined as ”PTM” and phosphorylation on serine, threonine, and tyrosine was allowed as additional variable modification. ”Label minimum ratio count” was set to 1. For the processing of search results, hits to the reverse and human contaminant databases were removed. Intensities in the different TMT channels were normalized based on total summed intensities of non-phosphorylated peptides that were quantified in all three replicates of both conditions. The Perseus software suite (v.1.6.6.1) was utilized to perform two-sided, unpaired t tests using log-transformed phosphosite and protein intensities and requiring a quantification for at least two out of the three replicates. Significant hits were determined specifying a constant S0 which was calculated for each dataset separately in R (version 3.4.1, function “samr”). It accounts for differing variances across the range of measured values and accordingly adapts the significance cut-off for statistical analyses. Significance levels were further corrected for multiple testing applying a permutation-based 5% FDR calculation.

#### Proteomic data analysis

The *de novo* network enrichment tool KeyPathwayMiner has been described (List et al., 2016). This used the murine STRING network (v. 11), filtered for regulatory interactions (post-translational modification, activation, or inhibition) of high confidence (combined score > 600). We augmented this network by adding known interactions of differentially phosphorylated tyrosine phosphoproteins curated in the IPA version 46901286 (between January and March 2019) database (experimentally observed, downstream, murine targets). In addition to this network, we subjected two datasets to KeyPathwayMiner: (a) differentially phosphorylated tyrosine phosphoproteins (absolute log2 ko/wt fold change of > 0.5 and p < 0.05) and (b) differential protein expression (absolute log2 fold change of > 0.5 and p < 0.05) with significant hits determined with a stringent 1% FDR. The largest subnetwork extracted by KeyPathwayMiner was enhanced with protein expression data and annotations using Cytoscape (Shannon et al., 2003, v. 3.7.1) and is shown in Figure 4B.

### Quantification and Statistical Analysis

Dot plots and statistical analyses were, unless specified differently, prepared and calculated with Prism 8.0 (Graphpad Software). Unpaired t tests were used to compare two groups and p < 0.05 was considered significant and p > 0.05 was considered not significant. Details for each experiment can be found in the respective figure legends.

## Supplemental Information

Document S1. Figures S1–S3 and Table S1

Document S2. Article plus Supplemental Information
