# Supplementary material for: PTPN2 Deficiency Enhances Programmed T Cell Expansion and Survival Capacity of Activated T Cells
Source: Cell Rep. 2020 Jul 28;32(4):107957. doi: 10.1016/j.celrep.2020.107957 (PMC7408006; doi:10.1016/j.celrep.2020.107957)
Supplement: Document S1. Figures S1–S3 and Table S1 [file mmc1.pdf]

Cell Reports, Volume *32*

Supplemental Information

PTPN2 Deficiency Enhances Programmed T Cell Expansion and Survival Capacity of Activated T Cells

Markus Flosbach, Susanne G. Oberle, Stefanie Scherer, Jana Zecha, Madlaina von Hoesslin, Florian Wiede, Vijaykumar Chennupati, Jolie G. Cullen, Markus List, Josch K. Pauling, Jan Baumbach, Bernhard Kuster, Tony Tiganis, and Dietmar Zehn

Fig. S1

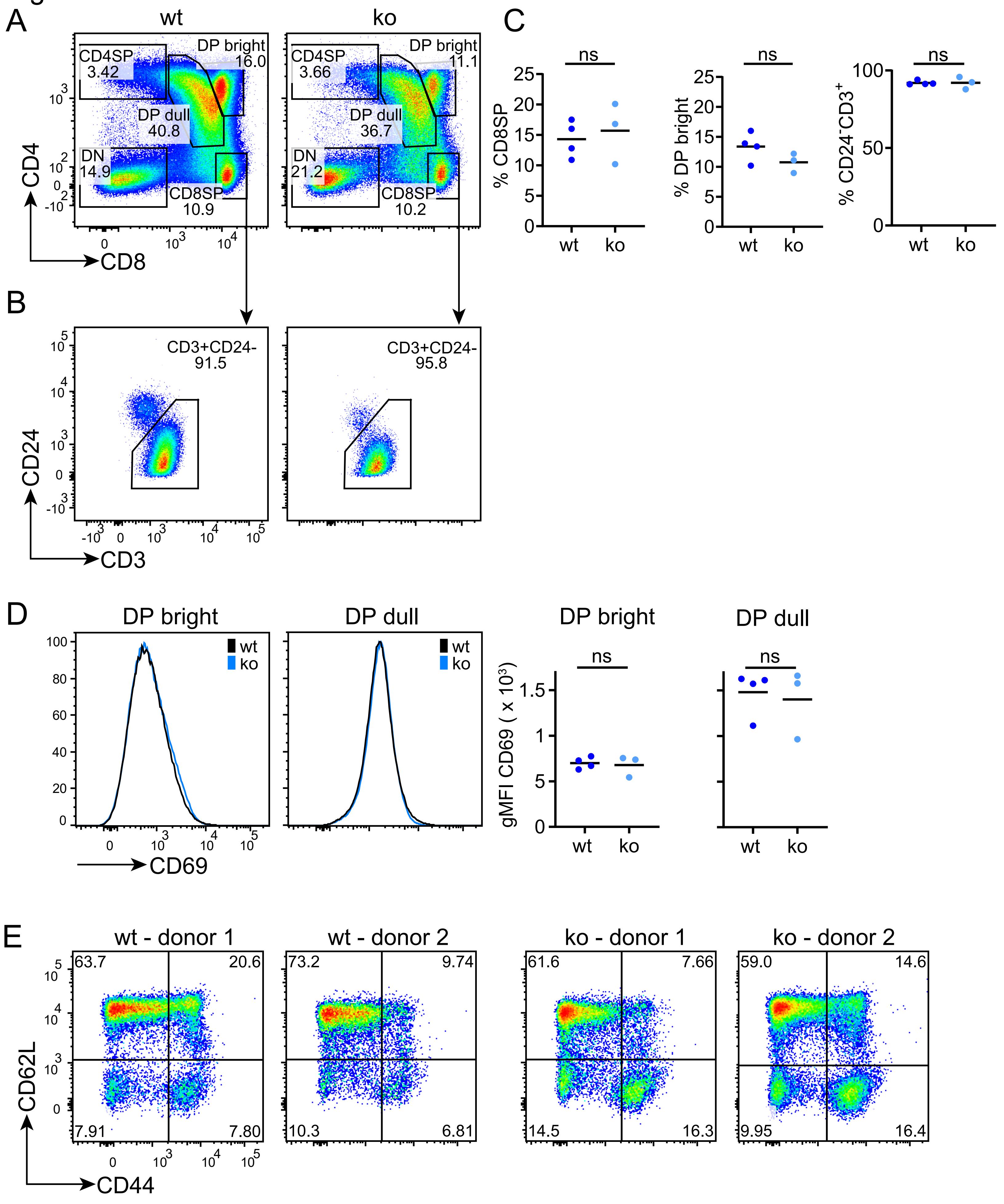
A
wt
ko
CD4SP
3.42
DP bright
16.0
DP dull
40.8
DN
14.9
CD8SP
10.9
CD4SP
3.66
DP bright
11.1
DP dull
36.7
DN
21.2
CD8SP
10.2
CD4
CD8
B
CD3+CD24-
91.5
CD3+CD24-
95.8
CD24
CD3
C
ns
% CD8SP
% DP bright
% CD24-CD3+
wt
ko
D
DP bright
DP dull
wt
ko
CD69
gMFI CD69 (x 10^3)
E
wt - donor 1
wt - donor 2
ko - donor 1
ko - donor 2
63.7
20.6
7.91
7.80
73.2
9.74
10.3
6.81
61.6
7.66
14.5
16.3
59.0
14.6
9.95
16.4
CD62L
CD44

Figure S1: Absence of PTPN2 does not impair thymic development. Related to Figure 1. The thymi of OT-I;*Lck*-Cre;*Ptpn2*$^{fl/fl}$ (ko) and OT-I;*Ptpn2*$^{fl/fl}$ (wt) mice were taken from donors at the age of 6-10 weeks. **A**) Frequencies of developing T cells among total thymocytes were determined by flow cytometry and FACS plots of the major developmental stages of two mice are shown. Data are representative of seven donor mice. SP: single positive, DP: double positive, DN: double negative **B**) The FACS plots show the percentage of CD24^{-}CD3^{+} cells of CD8-single positive cells. **C**) The dot plots show the summary of all 7 animals and each dot represents one individual mouse, horizontal lines the mean. **D**) The histograms show a representative overlay of the CD69 expression levels of the CD8^{+}CD4^{+} bright and dull populations and the dot plots show the geometric mean fluorescent intensity (gMFI) of all seven animals. **E**) The FACS plots show the pre-activation status of isolated OT-I CD8 T cells by means of CD44 and CD62L expression of 4 individual donor mice that were used in our experiments. Statistical analysis: unpaired t-test. ns (not significant) $p \geq 0.05$.

Fig. S2

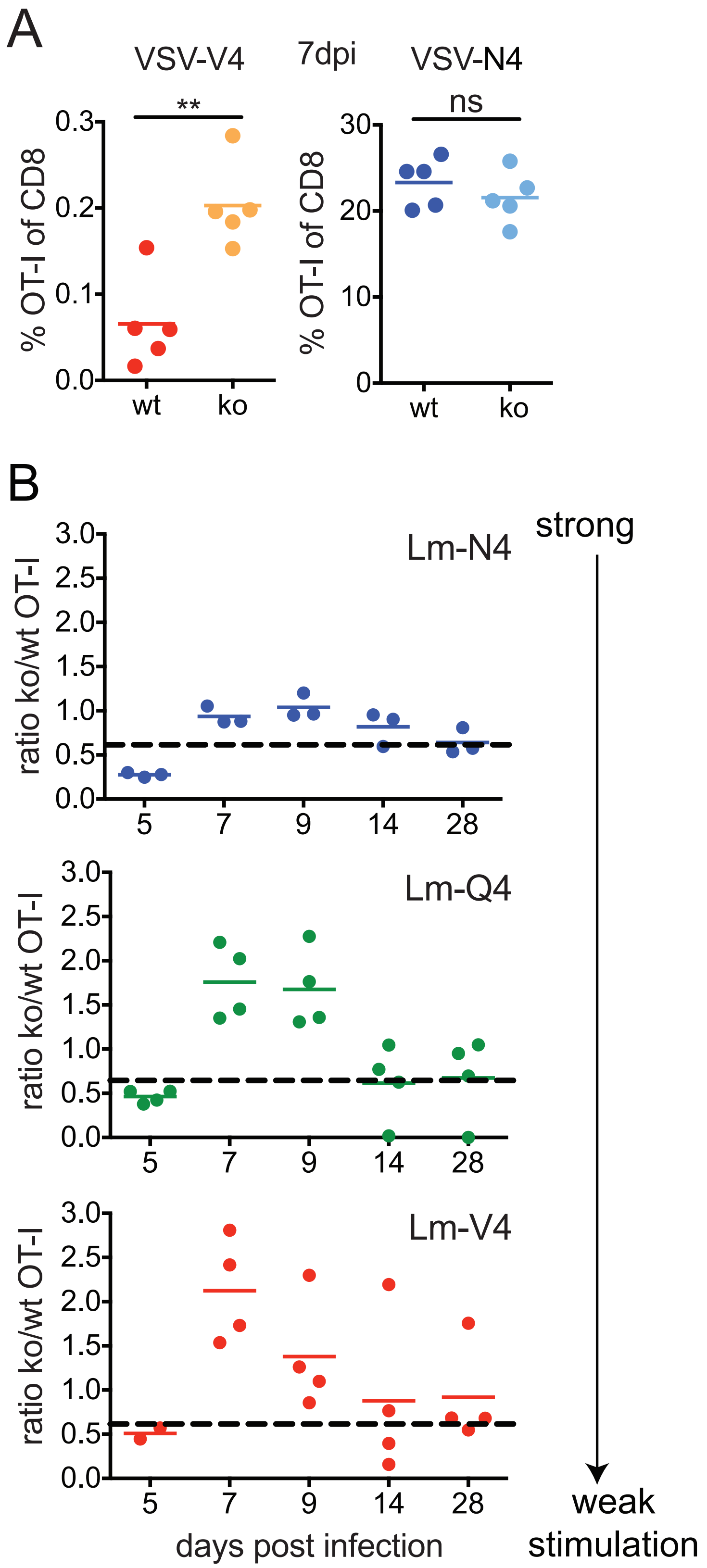
A
VSV-V4
7dpi
VSV-N4
**
ns
% OT-I of CD8
0.3
0.2
0.1
0.0
wt
ko
30
20
10
0
B
strong
Lm-N4
Lm-Q4
Lm-V4
ratio ko/wt OT-I
3.0
2.5
2.0
1.5
1.0
0.5
0.0
5
7
9
14
28
days post infection
weak
stimulation

Figure S2: Absence of PTPN2 delays the onset of T cell contraction. Related to Figures 1 and 2. CD45-congenic C57BL/6J host mice received 10^4 OT-I;*Lck*-Cre;*Ptpn2*$^{fl/fl}$ (ko) and OT-I;*Ptpn2*$^{fl/fl}$ (wt) CD8 T cells and were infected 24 hours later with ovalbumin-expressing pathogens. **A**) Frequencies of OT-I T cells among total CD8 T cells in the spleen were determined by flow cytometry 7 days after infection with 2 x 10^6 PFU VSV-V4 or VSV-N4. Data are representative of three independent experiments, with at least four mice per group. **B**) Plots show the ratio of ko/wt OT-I T cells at different time-points following infection with 1000 CFU Lm-N4 (SIINFEKL), Lm-Q4 (SIIQFEKL), or Lm-V4 (SIIVFEKL), which provide high, intermediate or low avidity ligands for OT-I T cells. The dashed line represents the initial engraftment ratios determined 1 day after transfer of a higher amount of wt or ko OT-I T cells of the same mixture in uninfected hosts. The data are representative of two independent experiments with 3-5 mice. Dots in all panels represent one individual mouse, horizontal lines the mean. Statistical analysis: unpaired t-test. $^{**}p \leq 0.001$, ns (not significant) $p \geq 0.05$.

Fig. S3

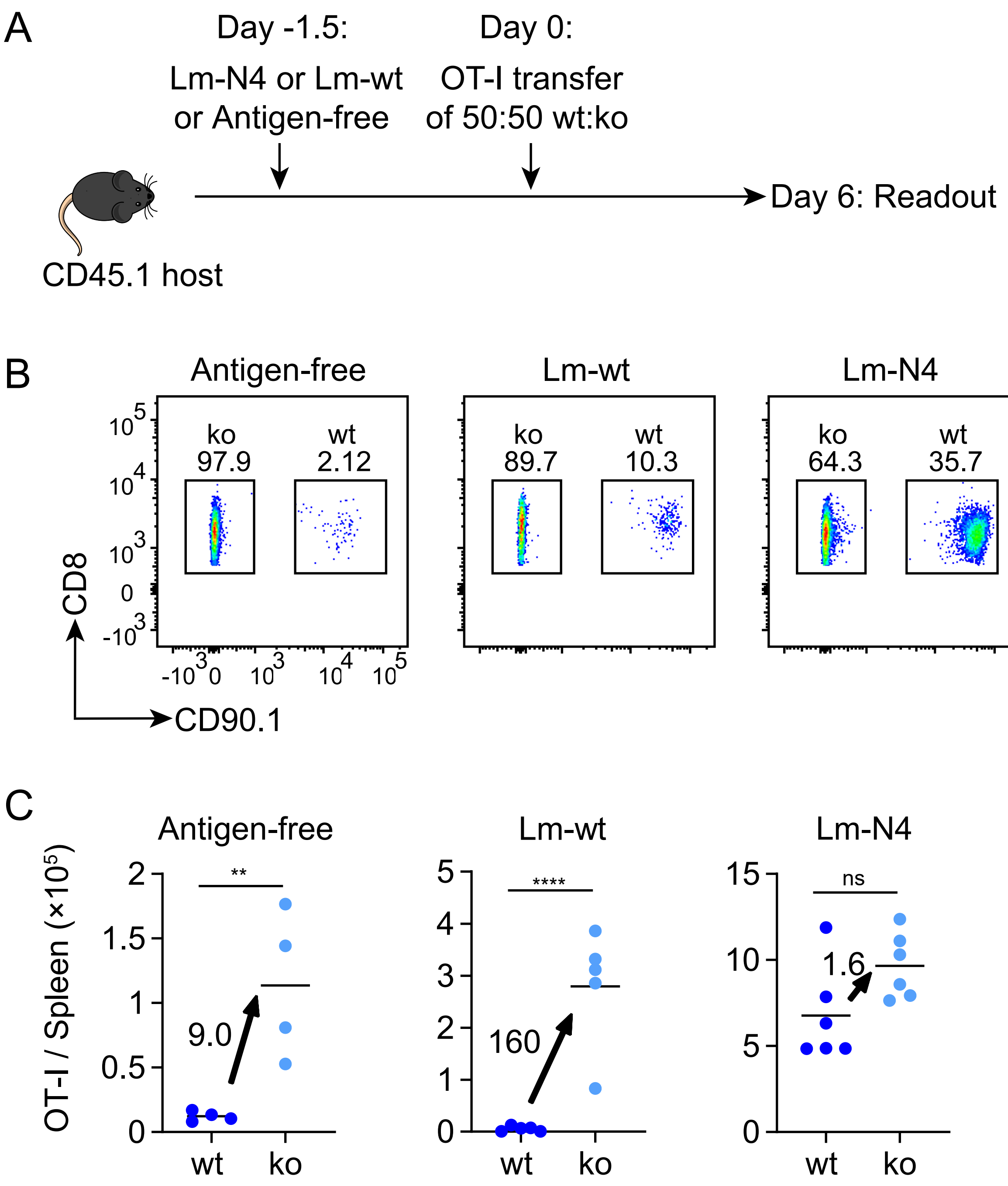
A
Day -1.5:
Lm-N4 or Lm-wt
or Antigen-free
Day 0:
OT-I transfer
of 50:50 wt:ko
Day 6: Readout
CD45.1 host
B
Antigen-free
Lm-wt
Lm-N4
ko
97.9
wt
2.12
ko
89.7
wt
10.3
ko
64.3
wt
35.7
CD8
CD90.1
C
Antigen-free
Lm-wt
Lm-N4
OT-I / Spleen (×10^5)
**
****
ns
9.0
160
1.6
wt
ko

Figure S3: Pre-activated PTPN2-deficient T cells expand significantly in response to antigen. Related to Figure 3. **A**) CD45-congenic C57BL/6J host mice were infected with 1000 CFU Lm-N4 or Lm-wt or kept antigen-free for 36 hours before receiving 5×10^4 OT-I;*Lck*-Cre;*Ptpn2*$^{fl/fl}$ (ko) mixed with 5×10^4 OT-I;*Ptpn2*$^{fl/fl}$ (wt) CD8 T cells that both had been activated *ex vivo* for 36 hours. **B**) Numbers of OT-I T cells in the spleen were determined by flow cytometry 7.5 days after infection and representative FACS plots of one mouse of each condition are displayed. **C**) The dot plots show the absolute numbers of OT-I T cells per spleen and each dot represents one individual mouse, horizontal lines the mean.

Data are representative of two independent experiments for the transfer in antigen-free hosts and one experiment for the transfer in infected hosts, with at least four mice per group. Statistical analysis: unpaired t-test. ****$p \leq 0.00001$, **$p \leq 0.001$, *$p \leq 0.01$, ns (not significant) $p \geq 0.05$.

Supplementary Table 1. Differentially phosphorylated tyrosine sites of PTPN2-deficient versus wt OT-I T cells. Related to Figure 4.

Proteins	Isoforms	Positions within isoforms	Amino acid	log2 ko/wt	Localization probability	Score
Stat3	P42227-3	704	Y	2.4	1.00	115
Stat3	P42227;B7ZC18;P42227-2	705;679;705	Y	2.3	1.00	138
Stat5b	P42232	699	Y	2.2	1.00	122
Stat5a	P42230	694	Y	1.9	1.00	108
Stat4	Q3V157;P42228	693;694	Y	1.4	0.99	116
Vav1	P27870;E9PXI0;Q8VDU4	844;820;805	Y	1.2	0.90	51
Ptpn18	Q3V441;Q61152	32;381	Y	1.2	0.77	119
Fyb	O35601;O35601-2	559;559	Y	1.1	1.00	122
Ptpn6	P29351;P29351-2;P29351-3;G3UYY5	564;566;525;64	Y	1.0	1.00	108
Mapk1	P63085	185	Y	1.0	1.00	190
Pdap1	Q3UHX2	70	Y	1.0	0.93	120
Srsf1	H7BX95;Q6PDM2	202;202	Y	0.9	0.98	98
Hist1h4a	P62806	52	Y	0.9	1.00	118
Pabpc1	P29341	364	Y	0.8	1.00	90
Itsn2	E9QNG1;B2RR82;Q9Z0R6;Q9Z0R6-2;A0A1W2P775	922;949;922;922;481	Y	0.8	1.00	138
Cd28	P31041	189	Y	0.7	1.00	165
Cblb	Q3TTA7;B9EKI5;Q3TTA7-2	889;845;737	Y	0.6	1.00	70
Cfl1	P18760;F8WGL3	68;68	Y	-0.7	0.87	179

The OT-I CD8 T cells were activated for 30 hours *in vitro* with anti-CD3/anti-CD28 coupled beads and analyzed via mass spectrometry. The results were filtered for a combined MS-score of > 50 and a localization probability of > 0.75.
